# Host and viral determinants for MxB restriction of HIV-1 infection

**DOI:** 10.1186/s12977-014-0090-z

**Published:** 2014-10-25

**Authors:** Kenneth A Matreyek, Weifeng Wang, Erik Serrao, Parmit Kumar Singh, Henry L Levin, Alan Engelman

**Affiliations:** Department of Cancer Immunology and AIDS, Dana Farber Cancer Institute and Department of Medicine, Harvard Medical School, Boston, MA 02215 USA; Section on Eukaryotic Transposable Elements, Program in Cellular Regulation and Metabolism, Eunice Kennedy Shriver National Institute of Child Health and Human Development, National Institutes of Health, Bethesda, MD 20892 USA; Present address: Department of Genome Sciences, University of Washington, Seattle, WA 98195 USA

**Keywords:** Innate immunity, MxB, Mx2, HIV-1, Restriction factor

## Abstract

**Background:**

Interferon-induced cellular proteins play important roles in the host response against viral infection. The Mx family of dynamin-like GTPases, which include MxA and MxB, target a wide variety of viruses. Despite considerable evidence demonstrating the breadth of antiviral activity of MxA, human MxB was only recently discovered to specifically inhibit lentiviruses. Here we assess both host and viral determinants that underlie MxB restriction of HIV-1 infection.

**Results:**

Heterologous expression of MxB in human osteosarcoma cells potently inhibited HIV-1 infection (~12-fold), yet had little to no effect on divergent retroviruses. The anti-HIV effect manifested as a partial block in the formation of 2-long terminal repeat circle DNA and hence nuclear import, and we accordingly found evidence for an additional post-nuclear entry block. A large number of previously characterized capsid mutations, as well as mutations that abrogated integrase activity, counteracted MxB restriction. MxB expression suppressed integration into gene-enriched regions of chromosomes, similar to affects observed previously when cells were depleted for nuclear transport factors such as transportin 3. MxB activity did not require predicted GTPase active site residues or a series of unstructured loops within the stalk domain that confer functional oligomerization to related dynamin family proteins. In contrast, we observed an N-terminal stretch of residues in MxB to harbor key determinants. Protein localization conferred by a nuclear localization signal (NLS) within the N-terminal 25 residues, which was critical, was fully rescuable by a heterologous NLS. Consistent with this observation, a heterologous nuclear export sequence (NES) abolished full-length MxB activity. We additionally mapped sub-regions within amino acids 26–90 that contribute to MxB activity, finding sequences present within residues 27–50 particularly important.

**Conclusions:**

MxB inhibits HIV-1 by interfering with minimally two steps of infection, nuclear entry and post-nuclear trafficking and/or integration, without destabilizing the inherent catalytic activity of viral preintegration complexes. Putative MxB GTPase active site residues and stalk domain Loop 4 -- both previously shown to be necessary for MxA function -- were dispensable for MxB antiviral activity. Instead, we highlight subcellular localization and a yet-determined function(s) present in the unique MxB N-terminal region to be required for HIV-1 restriction.

**Electronic supplementary material:**

The online version of this article (doi:10.1186/s12977-014-0090-z) contains supplementary material, which is available to authorized users.

## Background

Host organisms have developed a wide array of innate immunity proteins to prevent or mitigate viral infection. Expression of these proteins is often induced by interferon signaling, as their potent activities can come at the cost of host cell or organism viability. The study of retroviruses in particular has uncovered many examples of innate antiviral proteins, including apolipoprotein B mRNA editing enzyme, catalytic polypeptide-like (APOBEC) 3G [1], tripartite motif (TRIM) 5α [2] and TrimCyp [3], SAM domain and HD domain-containing protein (SAMHD) 1 [4,5], and bone marrow stromal cell antigen (BST) 2/Tetherin [6,7]. Recently, Myxovirus resistance protein 2 (MxB) was discovered to exhibit potent antiviral activity against HIV-1 infection [8-11].

In humans, MxB is one of two members of a family of dynamin-like large GTPases. Mx family proteins are found in almost all vertebrates, demonstrating their evolutionary importance for host organisms [12]. The X-ray crystal structure of human MxA showed that these proteins can be divided into three structurally folded domains: a globular GTPase domain, a largely C-terminal alpha helical stalk domain, and a series of alpha helices found in sequences adjacent to these domains which fold in the protein tertiary structure to form the bundle signaling element (BSE) [13]. Mx proteins also possess relatively unstructured N-terminal regions of varying length. These proteins readily multimerize into higher-order structures, with MxA known to form large ring-like assemblies [14,15].

Human MxA inhibits infection by a large number of negative-stranded RNA viruses, though it also counteracts other viral families [12]. The ability of MxA to inhibit a diverse set of viruses while specifically targeting different proteins across these families is atypical amongst innate immune proteins [16], suggesting an antiviral mechanism distinct from those of previously discovered restriction factors. Despite the breadth of known antiviral activities of MxA, little is known about the antiviral potential of MxB. Until recently, MxB was not thought to possess antiviral activity, which suggested that its purpose was solely to function during cellular nucleo-cytoplasmic transport [17]. In contrast to MxA, which is cytoplasmic, MxB localizes to the nuclear rim [18], which may play into its distinct pattern of antiviral activity.

A large-scale screen assessing the activities of interferon stimulated gene products against a panel of viruses first uncovered an antiviral activity of human MxB against HIV-1 [8]. More recently, a series of papers found MxB to be a key component of the interferon-mediated response against the early steps of HIV-1 infection [9-11]. The reverse transcription complex wherein HIV-1 RNA is reverse transcribed into double stranded linear DNA carries a fraction of the virion capsid (CA) protein [19,20], and CA mutations can accordingly confer resistance to restriction by MxB [9-11]. The viral integrase (IN) protein processes the long terminal repeat (LTR) ends of the viral DNA to yield the integration-competent preintegration complex (PIC), which subsequently transports the viral DNA into the nucleus for IN-mediated integration [21]. Although the recent studies agreed that MxB expression potently inhibited the early phase of HIV-1 replication including integration, they differed in terms of where in the lifecycle infection was blocked. For example, the measured effect on 2-LTR-containing DNA circles, which is utilized as a marker for PIC nuclear import [22], ranged from completely [9] or partially affected [11] to completely unaffected [10]. Additionally, initial results from these studies suggested MxB antiviral activity was independent of its GTPase activity [9,11], yet dependent on the inclusion of an N-terminal sequence harboring a nuclear localization signal (NLS) [11]. Here we clarify that MxB restricts PIC nuclear import as well as HIV-1 integration. Concordantly, our results confirm the critical nature of the N-terminal region NLS, which can be functionally exchanged by the heterologous basic-type NLS from simian virus (SV) 40 large T antigen.

## Results

### Experimental system

**Figure 1:**
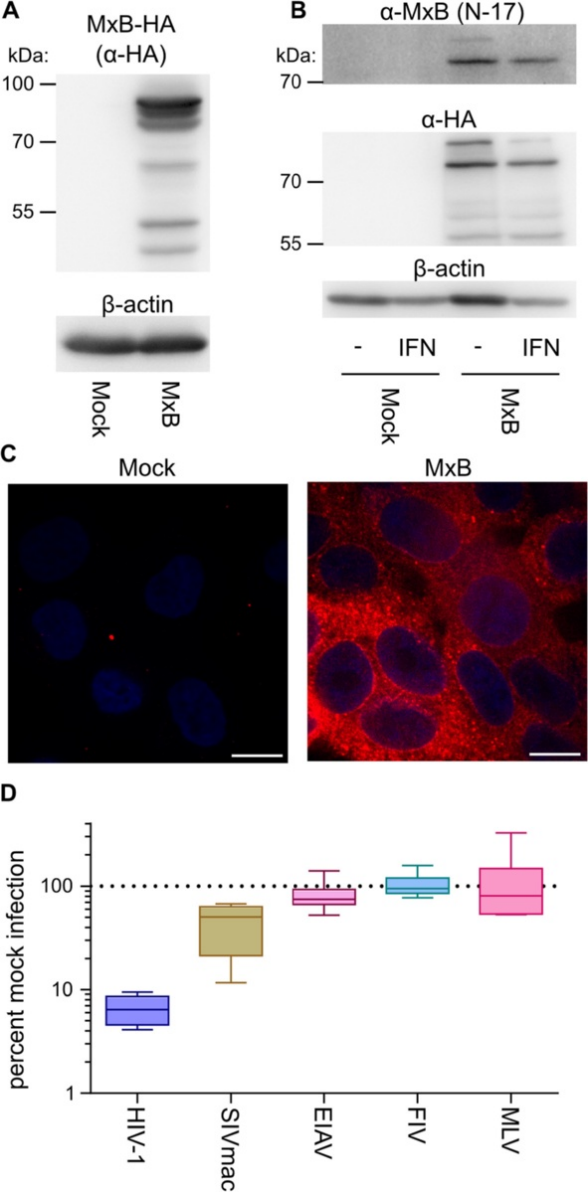
**MxB antiviral activities. (A)** Western blot of HOS cells expressing MxB-HA. β-actin was monitored to control for sample loading. **(B)** Western blot of untransduced or MxB-HA expressing HOS cell lysates extracted after treatment for 24 h with 1,000 U/ml of interferon α (IFN) as indicated. **(C)** Immunofluorescence microscopy of HOS cells stably expressing MxB-HA or mock-transduced cells. Blue-tinted ovals demarcate cell nuclei due to Hoescht 33342 staining of DNA. White horizontal bar, 10 μm. **(D)** Infection of MxB-HA expressing cells with various retroviral vectors plotted as percent infection of mock-transduced cells. Results are a summary of 6 independent experiments with error bars denoting 95% confidence intervals.

We tested the activity of MxB by stably expressing a C-terminally HA-tagged form in human osteosarcoma (HOS) cells (Figure 1A). These cells, which were previously used to dissect aspects of MxB antiviral activity [11], do not express appreciable endogenous MxB protein, either in the presence of absence of exogenously added interferon α (Figure 1B). Immunofluorescent staining of wild type (WT) MxB protein revealed that it localized to multiple compartments within HOS cells: a substantial fraction of MxB appeared as punctate signals within the cytoplasm, though additional staining could be observed along the nuclear rim (Figure 1C). Punctate cytoplasmic localization, which has been reported previously [23], may reflect the expression of N-terminal truncation products that presumably lack the N-terminal NLS [23] (Figure 1A) and/or protein aggregation. MxB potently restricted HIV-1 under these conditions (*P* <0.001), reproducibly inhibiting infection greater than 10-fold (Figure 1D). MxB also restricted SIVmac (*P* ~0.002), though the magnitude of this effect, ~2.6-fold, was significantly less robust than its activity against HIV-1. MxB was not significantly active against the more divergent lentiviruses equine infectious anemia virus (EIAV) and feline immunodeficiency virus (FIV), or the gammaretrovirus Moloney murine leukemia virus (MLV). Many of our subsequent antiviral studies accordingly employed EIAV and FIV as negative controls (see below).

### Correlation of MxB restriction with CA-targeting factors

**Figure 2:**
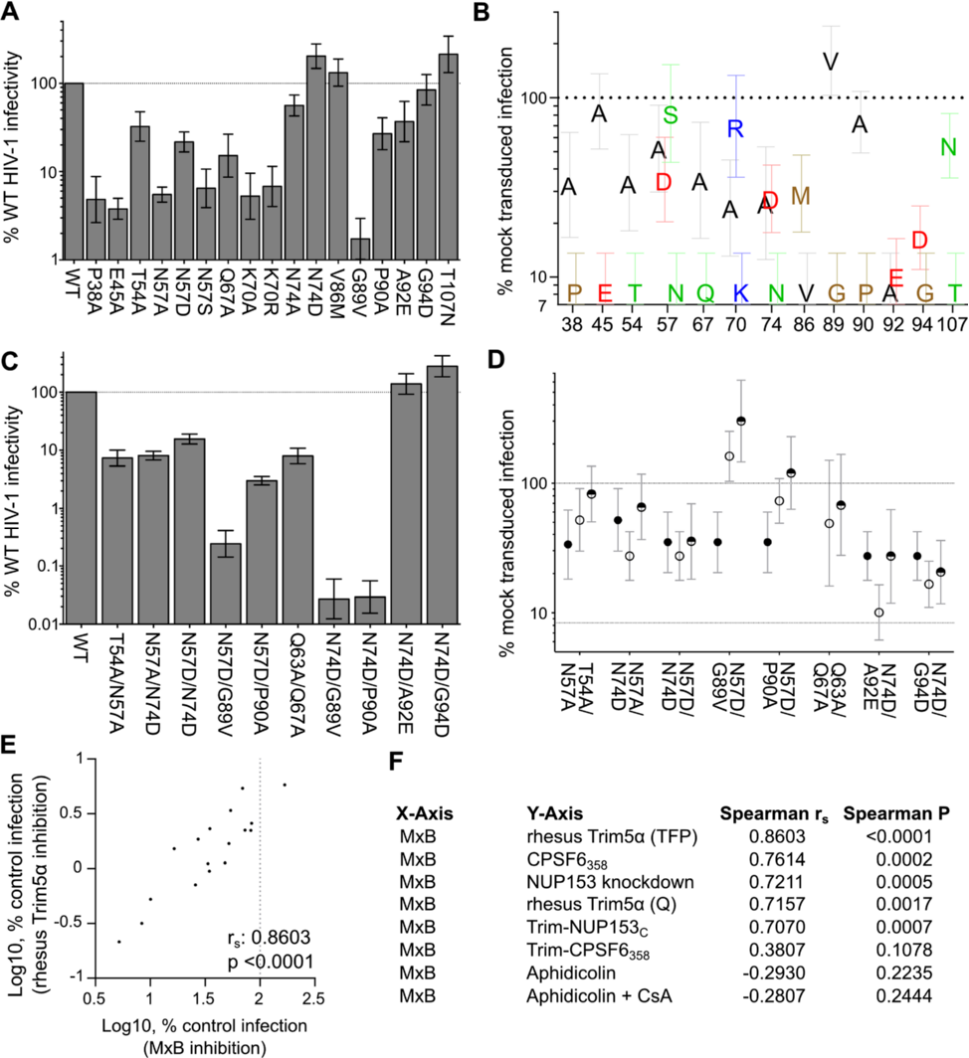
**HIV-1 CA and sensitivity to MxB restriction. (A)** Infectivities of CA mutant viruses normalized to the WT by input level of exogenous RT activity and expressed as percent of WT HIV-1 infection. **(B)** Sensitivity of the 17 CA single mutant viruses to MxB-HA, expressed as percent infection versus mock-transduced cells. The single letter codes along the bottom of the graph represent the WT residue at that position. **(C)** Normalized infectivities of 10 CA double mutant viruses relative to the WT. **(D)** Sensitivity of HIV-1 CA double missense mutant viruses (half black, half white filled circles) as compared to the parental single mutant viruses. Q63A alone was not tested. **(E)** Scatterplot comparing the sensitivity of CA missense mutant viruses to MxB-HA restriction with sensitivity to the rhesus TRIM5α TFP allele. **(F)** Pairwise comparison summary of MxB sensitivity with various CA-related manipulations of host cells, with Spearman rank correlation and *P* values denoted; see Additional file 1: Figure S1 for the corresponding scatterplots. Panel A-D results are averages of at least 6 independent experiments, with error bars denoting 95% confidence intervals.

The CA protein, which is the HIV-1 factor that primarily mediates PIC nuclear import [24], was previously found to determine the sensitivity of HIV-1 to MxB [9-11]. We assessed the sensitivities of a panel of 17 HIV-1 CA single missense mutant viruses that were previously used to investigate the mechanism of nucleoporin (NUP) 153 utility, and hence PIC nuclear import [25,26], to MxB restriction. The inherent infectivities of the mutant viruses spanned a relatively large range, from ~2% of the WT virus for the G89V mutant to ≥100% of WT for N74D, V86M, and T107N (Figure 2A). Consistent with the prior reports, numerous mutants, most notably E45A, N57A, N57S, K70R, G89V, P90A, and T107N, were significantly less sensitive than the WT virus to MxB restriction (*P* <0.02) (Figure 2B). In some cases, most notably for T54A/N57A and N57D/P90A, the combination of mutations yielded double mutant viruses that displayed less sensitivity to MxB restriction as compared to the parental mutants (Figure 2C and D).

We next compared the MxB sensitivity profiles of the CA mutant viruses to similar profiles generated when HIV-1 infection was perturbed through other means, including restriction by TRIM5α or a truncated version of cleavage and polyadenylation specific factor (CPSF) 6 (CPSF6_358_), or by cell growth arrest (Figure 2E and F) [26]. We found that the pattern of HIV-1 CA mutant virus resistance to MxB correlated strongly with those resistant to rhesus TRIM5α (Spearman *P* <0.0001 for the TFP allele; *P* = 0.0017 for the Q allele). There were also significant correlations in CA mutant sensitivity to CPSF6_358_-mediated restriction, NUP153 depletion, and restriction by the artificial Trim-NUP153_C_ fusion protein that harbors the CA-interacting C-terminal region of NUP153 (NUP153_C_) [26]. In contrast, there was only a weak correlation with restriction by the Trim-CPSF6_358_ fusion protein, and a weak negative correlation with sensitivity to growth arrest (Figure 2F and Additional file 1: Figure S1).

### IN activity as a secondary resistance determinant of MxB restriction

**Figure 3:**
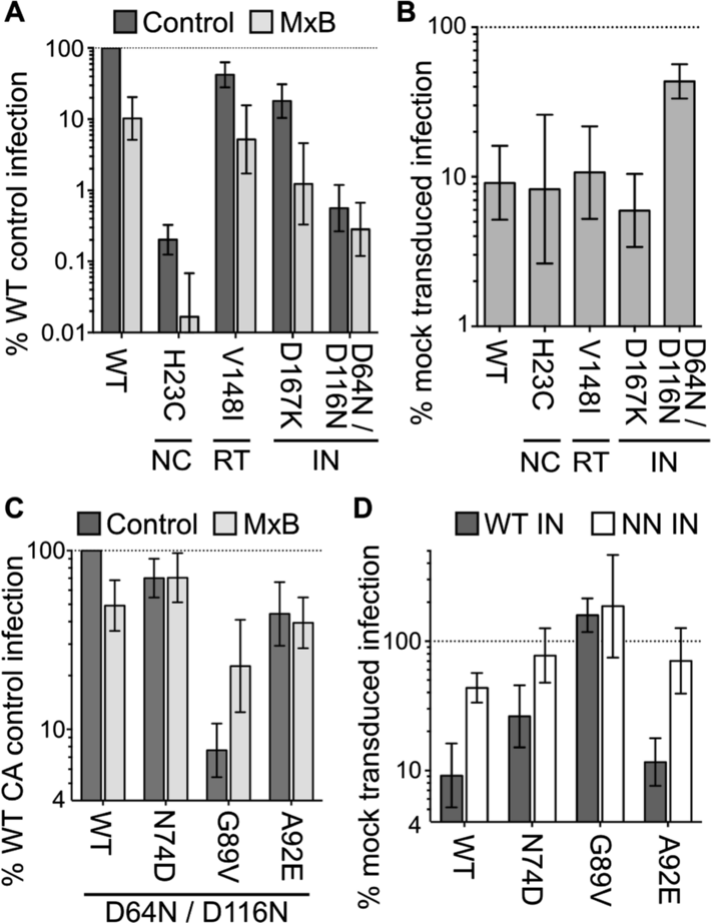
**Partial resistance of D64N/D116N IN active site mutant virus to MxB restriction. (A)** Levels of NC mutant H23C, RT mutant V148I, and IN mutant D167K and D64N/D116N infectivities, normalized to the WT based on input levels of exogenous RT activity, in control (dark grey) versus MxB-expressing (light grey) cells. Results are plotted relative to the WT virus in control cells (set at 100%). **(B)** The re-plot of panel A results highlights the infectivities of the indicated viruses in MxB-expressing cells relative to control cells. **(C)** RT-normalized levels of D64N/D116N IN mutant infectivities with versus without additional CA mutations in control (dark grey) versus MxB-expressing (light grey) cells. The infectivity of the WT CA, D64N/D116N IN mutant virus in control cells was set to 100%. **(D)** Extent of MxB restriction of WT and CA mutant viruses that carry WT (dark grey) or D64N/D116N (NN) mutant IN (white bars). Results are an average of 5 independent experiments, with error bars denoting 95% confidence intervals.

The details of where MxB restricted HIV-1 infection differed among the initial set of reports. Although all three papers found reverse transcription to be unaffected, Goujon et al. [9] concluded that MxB restricted PIC nuclear import whereas Liu et al. [10] reported that restriction occurred at the downstream step of integration. We utilized a number of assays, including quantitative (q) PCR readouts of reverse transcription, nuclear import, and integration, to address this issue (see below). To start, we assessed the sensitivity of different IN mutant viruses to MxB restriction. Although integration requires functional IN activity, IN active site mutant viruses such as D64N/D116N can support transient, low level (~1-2% of WT) HIV-1 infection in the absence of functional integration [25,27]. If PIC nuclear import is inhibited without any downstream influence on intranuclear trafficking or integration, D64N/D116N would be as sensitive as the WT virus to MxB restriction. D64N/D116N however was restricted to a significantly lower extent than was WT HIV-1 (*P* ~2 × 10^−4^) (Figure 3A and B). By contrast, the D167K IN mutant, which relies on chromosomal DNA integration for its expression [28], was as sensitive as the WT virus to MxB restriction. Because D64N/D116N was significantly less infectious than D167K (0.6% of the WT versus 18%; Figure 3A), mutant viruses that carried alterations in genes other than IN or CA, yet displayed similar levels of inherent infectivity, were used as controls. The H23C nucleocapsid (NC) mutant, which is weakly infectious (~0.2% of WT) [29], as well as the V148I reverse transcriptase (RT) mutant [30] (~40% WT activity), were both as sensitive as the WT virus to MxB restriction (Figure 3A and B). The relative resistance of IN mutant D64N/D116N to MxB restriction was moreover observed over a relatively wide (250-fold) range of multiplicity of infection (Additional file 2: Figure S2). Consistent with the existence of multiple viral genetic determinants, resistance to MxB restriction from IN inactivation was combinatorial with the partial resistance conferred by the N74D CA mutation, yet was epistatic with the full CA resistance mutant G89V (Figure 3C and D).

### MxB restricts nuclear import and integration

**Figure 4:**
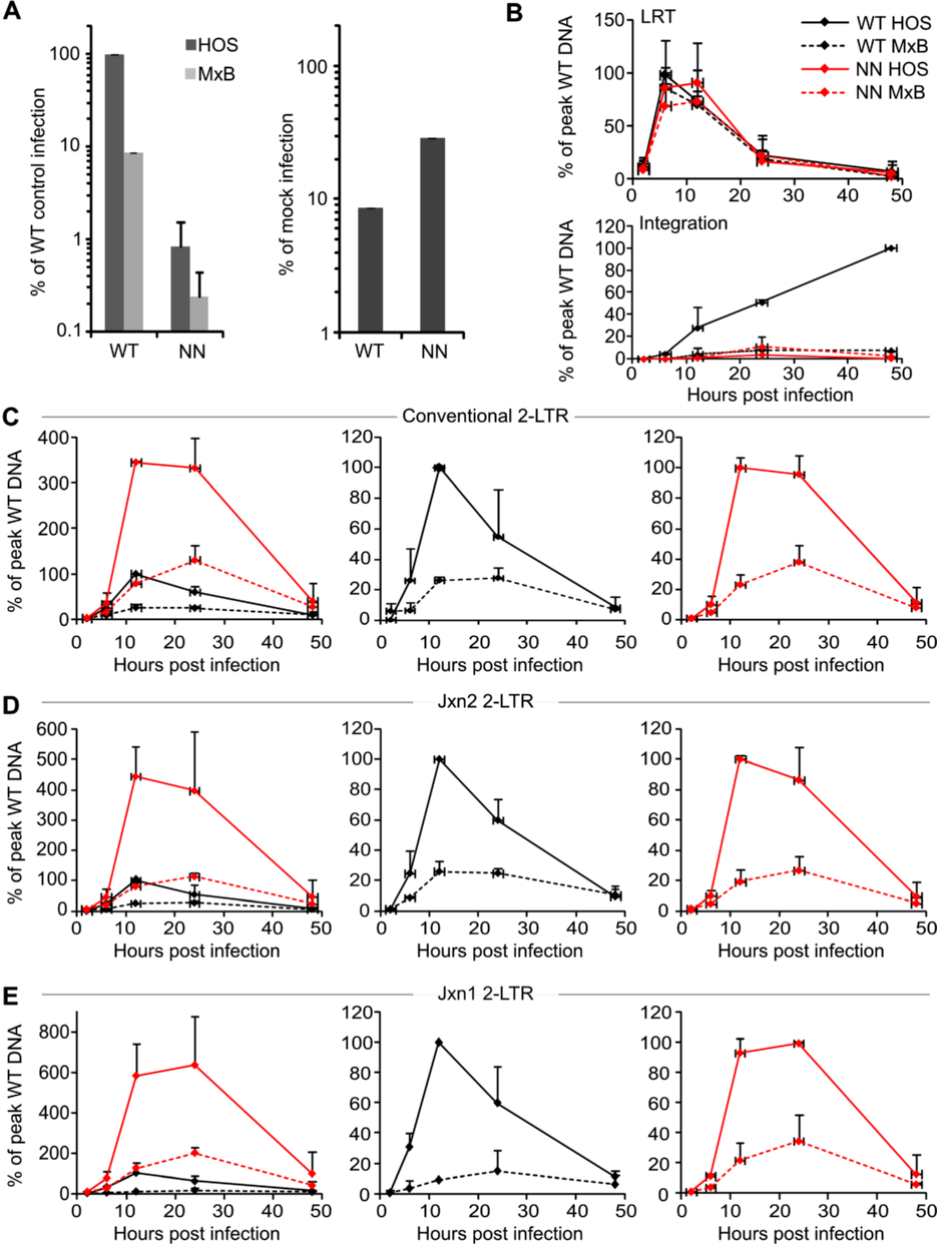
**WT and D64N/D116N IN mutant DNA metabolism in control and MxB-expressing cells.** **(A)** Levels of WT and D64N/D116N infectivities in control (dark grey) and MxB-expressing (light grey) HOS cells normalized for input RT cpm; the level of WT HIV-1 infection in control cells was set to 100% (left panel). Right panel, re-plot to highlight levels of WT and D64N/D116N IN mutant restriction by MxB. **(B)** Upper panel, WT and D64N/D116N (NN) late reverse transcription (LRT) products in control and MxB-expressing cells. WT and NN curves are black and red, respectively. Dashed lines, values from MxB-expressing cells. Lower panel, integration as assessed by *Alu*-R qPCR. **(C)** Levels of 2-LTR circles using conventional qPCR conditions. **(D and E)** Levels of 2-LTR circles using Jxn2 and Jxn1 qPCR conditions, respectively. The 4-member graphs to the left are split into two panels on the right in panels **C**-**E** to highlight the responses of WT (black lines) and NN mutant (red lines) viruses to MxB restriction (dashed lines). Results are an average of 2 independent experiments, with error bars denoting standard deviation (downward bars omitted to ease interpretation of coincident time points).

Levels of viral DNA intermediates at various times post-infection in control versus MxB-expressing cells were assessed by qPCR. PCR primers and probes were chosen to monitor late reverse transcription (LRT) products that form after the second template switch of viral DNA synthesis, 2-LTR circles that form in the cell nucleus, and HIV-1 integration [22,25,31,32]. The D64N/D116N mutant was analyzed alongside the WT virus to gain insight into the partial resistance conferred by IN inactivation. Under these conditions, infection by the WT and D64N/D116N viruses was restricted ~12 and 3.5-fold, respectively, by MxB (Figure 4A).

Consistent with previous reports [9-11], WT and IN mutant D64N/D116N viral reverse transcription was largely unperturbed by MxB expression (Figure 4B, upper panel). Different qPCR designs were utilized to assess levels of 2-LTR circles. Ligation of linear viral DNA ends that have not been processed by IN yields the novel circle junction sequence [33]. Because the responsible non-homologous DNA end joining (NHEJ) machinery resides in the nucleus, 2-LTR circles are used as a surrogate marker for PIC nuclear import [22,34]. HIV-1 however undergoes significant autointegration during infection, such that the processed LTR ends integrate into interior regions of the viral genome [35,36]. Because the kinetics of autointegration parallels that of viral DNA synthesis, autointegration can presumably transpire in the cell cytoplasm before PIC nuclear import [36]. Autointegration that occurs in the vicinity of the LTRs can score in 2-LTR PCR assays and accordingly cloud PIC nuclear import assessment [37]. De Iaco and Luban accordingly modified PCR assay conditions to take advantage of the unique circle junction sequence that forms through NHEJ in the cell nucleus. In one design, referred to here as Jxn2, the 3′ end of the reverse PCR primer harbors the nucleotides that are removed by IN prior to integration whereas in the other Jxn1 design, the Taqman probe spans the circle junction sequence [37].

Considering the ~12-fold block to HIV-1 infection, a comparatively modest decrease (~3.9-fold at peak levels) in the level of WT 2-LTR circles was observed in MxB-expressing cells using qPCR conditions that do not distinguish molecules that contain circle junction sequences from those that may arise from autointegration (Figure 4C, solid and dashed black lines). Jxn2 qPCR yielded this same differential whereas the 2-LTR circle defect modestly increased, to ~6.8-fold, using the Jxn1 design (Figure 4D and E, black lines). As expected, the D64N/D116N mutant virus supported the formation of significantly more 2-LTR circles than did the WT virus [38-40] (Figure 4C-E left panels, red and black solid lines). The IN mutant viral 2-LTR circle defect, measured as ~4.4, 3.8, and 2.9-fold at peak levels using conventional, Jxn2, and Jxn1 qPCR conditions, respectively, was roughly similar to that of the WT virus (Figure 4C-E, red lines). The level of WT HIV-1 integration in MxB-expressing cells was ~7.4% of the level achieved in control cells, which accounted for the 8.6% level of virus infection that was assessed through bulk luciferase activity (Figure 4B, lower panel and Figure 4A).

### MxB expression alters the distribution of integrated proviruses without affecting PIC integration activity

**Figure 5:**
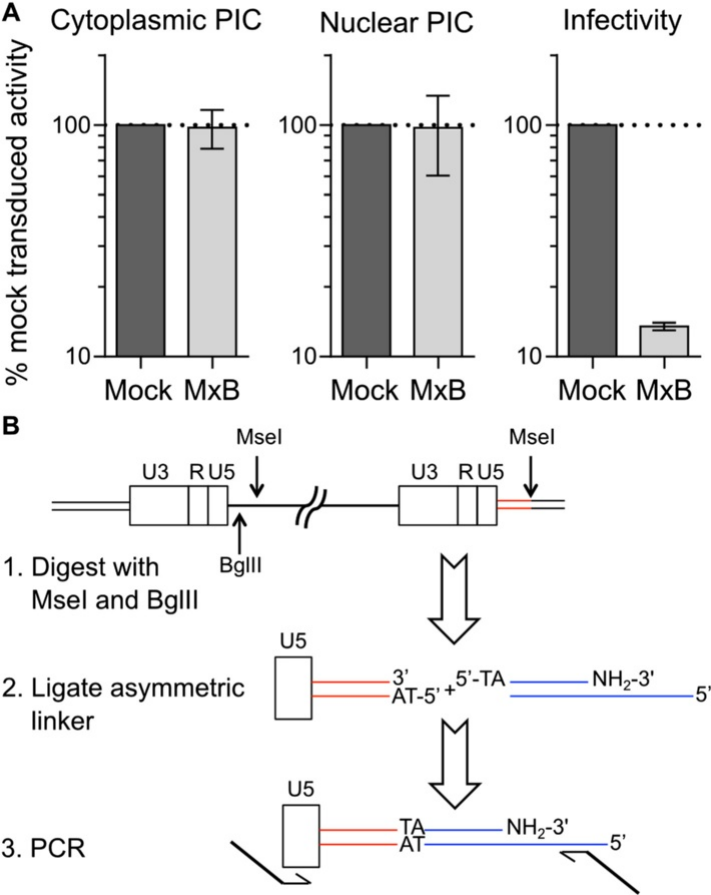
**PIC activity and integration site sequencing strategy. (A)** PICs extracted from the cytoplasm (left panel) or nucleus (center panel) of mock-transduced (dark grey bars) or MxB-expressing cells (light grey bars) were assessed for *in vitro* integration activity. Percent infectivity, determined 48 h after infection (right panel), revealed the level of MxB restriction under these infection conditions. Results are the average of 4 independent experiments, with error bars denoting standard error of the mean. **(B)** Integration site sequencing strategy. In the representative HIV-1 provirus the viral DNA internal to the LTRs is a single bold line and the abutting cellular DNA is two thin lines (the region to be sequenced is in red). Thin blue lines, asymmetric DNA linker. The bold extensions of PCR primers denote elements required for Illumina sequencing. HIV-1 DNA harbors numerous MseI sites; only the relevant site downstream from the upstream U5 sequence is shown.

The results of the previous experiments indicated that the defect in IN mutant D64N/D116N nuclear import, which was ~3.7-fold by averaging the results of the different 2-LTR qPCR assays, accounted for the 3.5-fold infectivity defect of this virus. By contrast, the 2-LTR circle defect of the WT virus did not seem to fully account for its infection or integration defect. Two additional experiments were therefore performed to further probe the effect of MxB expression on HIV-1 integration. We first measured the ability of PIC-associated IN to support the integration of endogenous viral DNA into heterologous target DNA *in vitro*, and subsequently assessed the distribution of integrated proviruses across the cellular genome. Cytoplasmic and nuclear PICs isolated from MxB-expressing cells notably supported similar levels of *in vitro* integration activities as those isolated from matched control cells (Figure 5A).

The distribution of HIV-1 integration sites was assessed using a ligation-mediated (LM)-PCR design modified to sequence viral U5-cellular DNA junctions on the Illumina platform (Figure 5B) [41,42]. In brief, cellular DNA isolated from infected cells was digested with the 4-bp cutter MseI and 6-bp cutter BglII; BglII was included to suppress amplification of the MseI site that lies downstream from the internal copy of U5 in the upstream LTR. The digested DNA was ligated to an asymmetric linker containing a 5′-TA overhang, and the ligation products were amplified by PCR using primers that annealed to U5 and linker DNA. A key modification here was the inclusion of heterologous sequences required for sequencing, including Illumina P5 and P7 adapters, in the PCR primers. Hence, only one round of PCR amplification was required prior to sequencing. The resulting sequences were parsed for U5 and linker DNA content, compared to human genome build 19 (hg19), and annotated for features such as genes, transcription start sites (TSSs), CpG islands, and gene density. Products of HIV-1 autointegration, ambiguous cell DNA reads, and duplicated integration sites were omitted from the bioinformatics analysis.

Control HOS cells yielded 476,305 unique HIV-1 integration sites whereas 50,065 were determined from MxB-expressing cells (Table 1). As expected [41], HIV-1 greatly favored integration into genes: 63.6% of control cell integrations occurred within RefSeq genes whereas 36.1% was the value expected based on a matched random control (MRC) dataset of 476,280 computer-generated sites. In the presence of MxB integration into genes fell off somewhat, to 58.0% of the sites. This value was statistically different from both the control HOS cell and MRC numbers (Table 1).

**Table 1:** **Effects of MxB restriction on HIV-1 integration site preferences** ^**a**^

**Library**	**Unique sites**	**Within Refseq genes (%)**	**Within 5 kb (+/− 2.5 kb) of TSS (%)**	**Within 5 kb (+/− 2.5 kb) of CpG island (%)**	**Average gene density within 1 Mb (+/− 0.5 Mb) of integration sites** ^**b**^
HOS cells	476 305	302 900 (63.6)^c^	14 814 (3.11)^d^	13 737 (2.88)^e^	19.3
HOS-MxB cells	50 065	29 041 (58.0)^f^	1 126 (2.25)^g^	986 (1.97)^h^	14.0
MRC	476 280	171 999 (36.1)	15 334 (3.22)	13 101 (2.75)	8.8

^a^Statistical comparisons performed by Fisher’s exact test.

^b^Based on complete genes. See Figure 6C for statistical analysis.

^c^ *P* value versus MRC, <2.2 × 10^−308^; versus HOS-MxB cells, 4.4 × 10^−132^.

^d^ *P* value versus MRC, 0.002; versus HOS-MxB cells, 2.6 × 10^−35^.

^e^ *P* value versus MRC, 8.4 × 10^−5^; versus HOS-MxB cells, 3.5 × 10^−35^.

^f^ *P* value versus MRC, <2.2 × 10^−308^.

^g^ *P* value versus MRC, 2.6 × 10^−35^.

^h^ *P* value versus MRC, 7.4 × 10^−27^.

**Figure 6:**
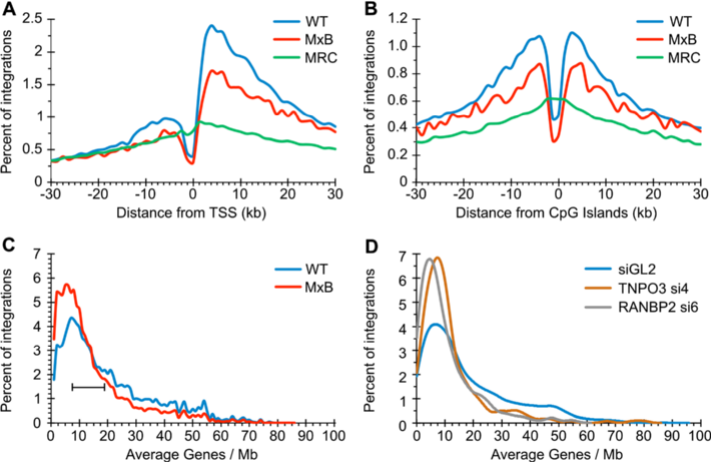
**Integration patterns in control versus MxB-expressing cells.** **(A)** Number of integration sites counted in 1.25 kb bins (x-axis ticks) were plotted as percent of total from 30 kb upstream (negative x-axis value) to 30 kb downstream of TSSs. Blue and red lines, data from control and MxB-expressing HOS cells, respectively; The green plot, MRC values. The sites ~4 kb to 12 kb upstream of TSSs with significantly greater levels of integration than random in WT HOS cells in the vast majority of cases also mapped within RefSeq genes, which was attributed to the presence of internal promoters in the human genome [44]. **(B)** Frequency of HIV-1 integration sites surrounding CpG islands. Line colorings are same as in panel **A**. See Additional file 3: Figure S3 for statistical analysis of panel A and B results. **(C)** Percent integration sites (y-axis) plotted against number of genes per Mb (x-axis) for infections conducted using control (blue line) and MxB-expressing (red line) HOS cells. Wilcox Rank-Sum test analysis of gene density targeting values (Table 1) yielded *P* values < 2.2 × 10^−302^ for all comparisons (WT versus MRC, MxB versus MRC, and WT versus MxB). Fisher’s exact test was therefore conducted on the subset of integration sites that fell between 8 and 19 genes per Mb (bracket). Herein, WT and MxB-expressing cells harbored 34.8% and 35.4% of all integrations, respectively; the MRC value was 30.2%. The WT versus MRC comparison yielded *P* <2.2 × 10^−308^. MxB-expressing cells versus MRC yielded *P =*2.8 × 10^−124^ whereas *P* =0.01 was determined for control versus MxB-expressing cells. **(D)** Same as in panel **C**, except the following data from Ocwieja et al. [45] was analyzed: control siGL2, 7,140 integration sites; TNPO3 si4, 3,923 sites; RANBP2 si6, 2,114 sites. TNPO3, transportin 3. The panel **D** graph was smoothed using kernal estimation [45] due to relatively fewer numbers of integration sites.

HIV-1 integration frequencies surrounding TSSs and CpG islands were initially determined for 5 kb (±2.5 kb) windows. As previously observed [43], the level of promoter proximal integration in control cells, 3.11%, was similar to the MRC value of 3.22% (Table 1). MxB expression modestly reduced the frequency obtained from control cells to the value of 2.25%. Likely due to our relatively large datasets, each of these differences was nevertheless statistically significant. To gain further insight into the effect of MxB restriction on the distribution of HIV-1 integration sites, provirus numbers within expanded 60 kb windows (30 kb upstream to 30 kb downstream of TSSs and CpG islands) were counted in 1.25 kb bins. The preference for HIV-1 to integrate into gene bodies was evident throughout the window downstream from TSSs (Figure 6A and Additional file 3: Figure S3A). By contrast, the virus sharply avoided regions ~1.25 kb immediately upstream from TSSs. MxB expression predominantly altered the control cell integration pattern from ~12.5 kb upstream to 25 kb downstream from TSSs, with the greatest effects occurring within the initial ~12 kb of gene regions (Figure 6A and Additional file 3: Figure S3A-C). The breakdown of CpG island targeting highlighted that HIV-1 avoids regions ~1.25 kb upstream of this annotation (Figure 6B and Additional file 3: Figure S3D). Symmetric peaks of integration preferences surrounding this “cold spot” were observed at ~6.25 kb upstream and 3.75 kb downstream from CpG islands. Although MxB expression did not grossly affect the overall pattern of CpG island sensing by the integration machinery, significant differences from control cells were observed from ~16.25 kb upstream to 15.0 kb downstream from CpG islands (Figure 6B and Additional file 3: Figure S3E and F).

As expected [41,46], HIV-1 additionally favored integration into chromosomal regions that were relatively enriched in genes: as compared to the calculated MRC value of 8.8 genes per Mb, the virus selected regions that on average harbored 19.3 genes per Mb of DNA in control cells. MxB expression significantly altered this preference, yielding an intermediate value of 14.0 genes per Mb (Table 1). Similar intermediate effects were previously noted when cell factors implicated in PIC nuclear import, for example transportin 3 or RANBP2, were depleted by RNA interference [45]. To gain insight into the consequences of MxB expression versus transportin 3 or RANBP2 depletion, percent integration sites were plotted against gene density. MxB expression yielded shifts in the gene density profile that overall appeared similar to the shifts elicited by nuclear transport factor depletion (Figure 6C and D).

### MxB GTPase active site residues

**Figure 7:**
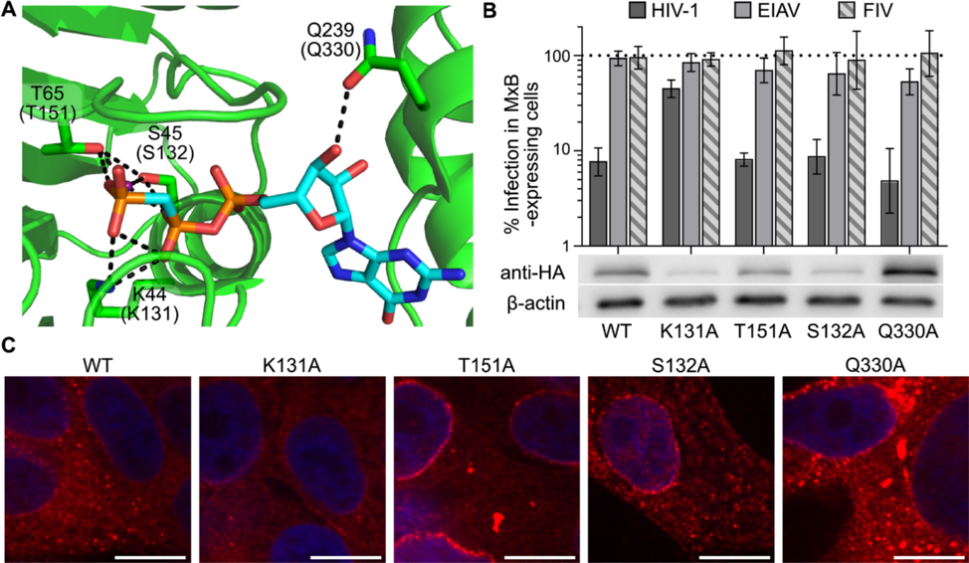
**Activities of MxB GTPase active site mutant proteins. (A)** X-ray crystal structure of dynamin active site with bound phosphomethylphosphonic acid guanylate ester (GCP) (pdb code 3zyc). Dynamin side chain contacts with GCP (cyan backbone) are labeled, with the corresponding conserved MxB residue labeled in parenthesis. Dotted black lines denote hydrogen bonds. Remaining colors: red, oxygen atoms; blue, nitrogen; orange, phosphorus. **(B)** (top) WT or MxB mutant protein activities against HIV-1 (dark grey), EIAV (light grey) or FIV infection (striped). Results are an average of at least 6 independent experiments, with error bars denoting 95% confidence intervals. (bottom) Representative western blots from at least 3 independent experiments. **(C)** Immunofluorescent microscopy of HOS cells expressing WT MxB or the indicated mutant protein. Each bar represents a distance of 10 μm.

We next focused on the characteristics of MxB required to restrict HIV-1 infection. We tested a moderate panel of 21 MxB deletion and missense mutant proteins, which exhibited a range of expression levels and HIV-1 restricting activity (summarized in Additional file 4: Figure S4). Among the tested mutations were those that targeted the MxB GTPase domain. The GTPase domain of MxB is highly related to other proteins derived from the dynamin family of large GTPases, such as MxA and dynamin itself. We found that dynamin side chains involved in GTP binding were conserved in MxB, suggesting functional importance (Figure 7A). Mutant forms of MxB harboring alanine residues at these positions were stably expressed in HOS cells (Figure 7B and C). With the exception of K131A, which was expressed at the lowest level among all tested mutants (Figure 7B and Additional file 4: Figure S4), the active site mutant MxB proteins restricted HIV-1 to levels indistinguishable from that of the WT protein (Figure 7B, dark gray bars). Though all GTPase mutants exhibited localization similar to WT MxB by immunofluorescence (Figure 7C), we infer that the dramatically reduced steady-state expression level of the K131A protein likely influenced its weakened restriction activity against HIV-1.

### The MxB loops

**Figure 8:**
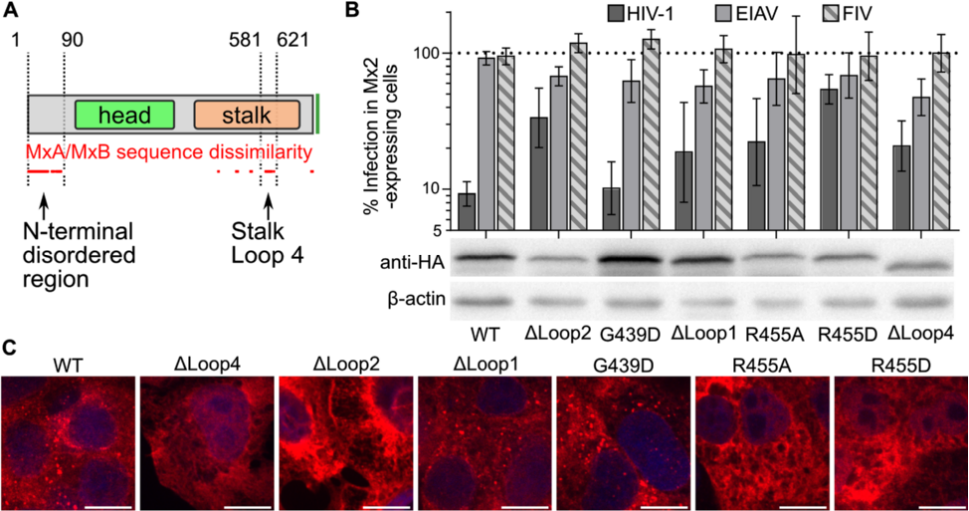
**MxB stalk mutants and antiviral activity. (A)** The MxB protein schematic highlights regions of sequence diversity from the human MxA protein; each red dot denotes an MxB position where 5 consecutive residues differed from MxA. **(B)** Antiviral activities of MxB stalk mutant proteins. Results are an average of at least 5 independent experiments, with error bars denoting 95% confidence intervals. **(C)** Representative immunofluorescent microscopy results; white bar, 10 μm. See Additional file 4: Figure S4 for results of quantitative western blotting.

Human MxA and MxB are highly related proteins (~56% amino acid identity; ~70% amino acid similarity). Two areas that exhibit the greatest extent of dissimilarity between the two proteins are the N-terminal regions that precede the first helix of the BSE (lengths of ~42 and ~90 residues in MxA and MxB, respectively) and Loop4 of the stalk domain (lengths of ~38 and ~40 residues in MxA and MxB, respectively) (Figure 8A). Because Loop4 is an important specificity determinant of MxA inhibition of orthomyxoviruses [47], we assessed the importance of MxB Loop4 in restricting HIV-1 by replacing the 32 amino acids that span residues 584–615 with a short flexible stretch of unrelated sequence (GAGAG). Despite the relative large disruption of endogenous peptide sequence, this construct retained fairly robust activity, inhibiting HIV-1 infection by ~5-fold (Figure 8B). Loop4 was not resolved within the crystal structures of MxA [13,48], but appears to influence MxA multimerization by making contacts with adjacent subunits within a multimer [48]. In fact, though many of the loops present in the stalk of dynamin superfamily proteins are involved in functional oligomerization, none of these regions was absolutely required for MxB antiviral activity. Stalk Loop2 is required for MxA oligomerization [13], but its removal only modestly reduced the restriction activity of the mutant protein (~3-fold as compared to WT MxB; *P* <0.0001). Deletion of Loop1 similarly reduced MxB antiviral activity by only ~2-fold. MxB mutant G439D corresponds to yeast Dnm1 assembly mutant G385D [49], yet the G439D change did not noticeably effect MxB antiviral activity. Lastly, the R408D mutation in MxA reduced oligomerization and antiviral activity in an Influenza A minireplicon system [48]. The corresponding MxB residue (Arg455) appeared to effect HIV-1 restriction as well: whereas the R455A mutation only modestly reduced antiviral activity, the R455D change largely rendered MxB unable to inhibit HIV-1 (*P* <0.0001) (Figure 8B). Though the steady state level of R455D mutant MxB expression was less than the WT, it was expressed at similar levels as GTPase domain mutants S132A and T151A, which were both fully active (Additional file 4: Figure S4). The Loop4, Loop2, and Arg455 mutant proteins notably exhibited more diffuse intracellular staining than WT MxB, which we infer is due to altered oligomerization equilibria within the cell. Interestingly, the localization of the least active mutant, R455D, was most prominently perturbed, with visible localization within the nucleoplasm (Figure 8C).

### The MxB N-terminal region

**Figure 9:**
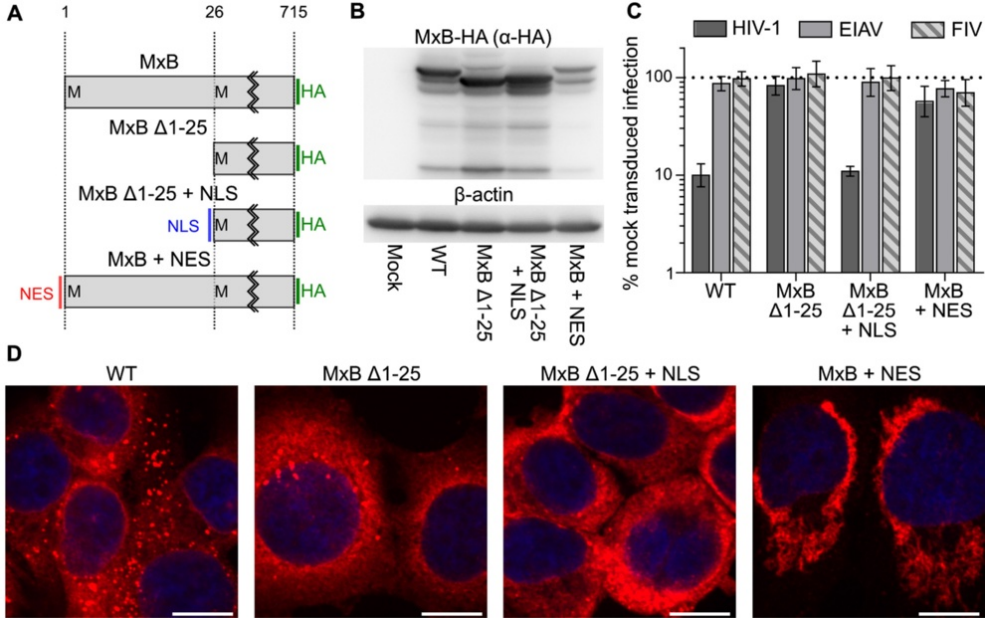
**Functional heterologous NLS and NES sequences. (A)** Schematic of MxB nuclear localization mutants. The location of heterologous NLS and NES sequences are denoted in blue and red, respectively; the common C-terminal HA tag is in green. Western blotting **(B)**, antiviral activity **(C),** and immunofluorescent microscopy **(D)** of WT MxB and mutant proteins. Results in panel C represent the geometric mean of at least 8 independent experiments, with error bars denoting 95% confidence intervals. The differences in HIV-1 restriction by MxB Δ1-25 or MxB + NES with WT MxB was highly significant (*P* <0.0001). The horizontal bars in panel D represent the distance of 10 μm.

The N-terminal 25 residues of MxB harbor a NLS that is necessary for MxB association with the nucleus [18]. It was previously noted that an N-terminal truncation mutant lacking these residues (Δ1-25) was inactive against HIV-1 [11]. We extended this analysis by testing two heterologous N-terminal localization mutants: we appended the basic NLS PKKKRKV from the SV40 large T antigen onto Δ1-25 (Δ1-25 + NLS) or added the nuclear export sequence (NES) derived from cAMP-dependent protein kinase inhibitor alpha onto the full-length protein (Figure 9A). The mutant proteins expressed similarly to WT MxB, except for MxB + NES, which was expressed ~2-fold less than the WT protein (Figure 9B and Additional file 4: Figure S4). Notably, the level of expression of MxB + NES was equal to or greater than those of highly active mutants T151A and S132A (Additional file 4: Figure S4). As previously observed, deletion of the initial 25 residues containing the endogenous NLS rendered MxB inactive against HIV-1 (Figure 9C). Appending the SV40 NLS onto this truncation protein however fully restored restriction. Furthermore, the NES-containing MxB protein was inactive (~1.7-fold reduction to HIV-1 infection, which did not differ significantly from EIAV and FIV controls). These results show that MxB localization, rather than the specific sequence of the N-terminal 25 residues, dictates MxB activity against HIV-1 infection. Obvious WT-like nuclear rim staining was not observed with either the Δ1-25 or Δ1-25 + NLS mutants (Figure 9D), though prominent perinuclear staining in the latter case confounded this assessment.

**Figure 10:**
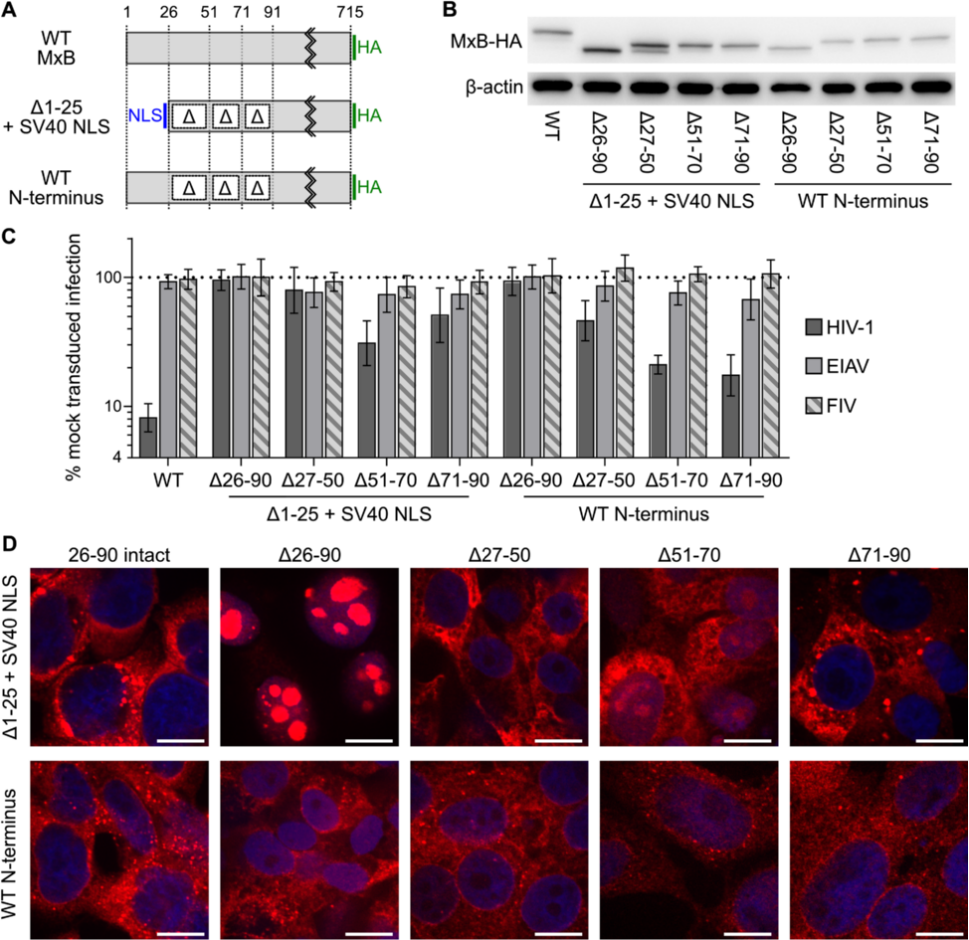
**Activity and localization of N-terminal deletion mutants.** Schematic **(A)**, western blotting **(B)**, antiviral activity **(C)**, and immunofluorescence microscopy **(D)** of N-terminal deletion mutants. Panel **C** results represent the geometric mean of at least 6 independent experiments, with error bars denoting 95% confidence intervals. All mutant proteins restricted HIV-1 infection to significantly lower levels than WT MxB (*P* <0.002). The bars in panel **D** represent 10 μm.

We next assessed the importance of the remaining sequence found within the unique N-terminal region of MxB. We first deleted these residues (26–90) from both active forms, i.e., from the full-length protein and from the Δ1-25 + NLS variant (Figure 10A). Both truncated forms were expressed similarly to the WT protein (Figure 10B and Additional file 4: Figure S4). Residues 26–90 of MxB were absolutely required for antiviral activity in both contexts (Figure 10C). Notably, Δ1-90 + NLS exhibited dramatically altered localization: the protein localized predominantly within the nucleus, with distinct nucleolar staining (Figure 10D, upper row). Lastly, we engineered a series of 20–25 amino acid deletions within residues 26–90 to narrow down specific regions of functional importance, again in the context of our two active WT and Δ1-25 + NLS forms (Figure 10A). Deletion of residues 27–50 abolished Δ1-25 + NLS antiviral activity and significantly crippled the activity of the construct with an intact N-terminus (~2.2-fold residual restriction activity). Deletions of amino acids 51–70 and 71–90 were far less disruptive, as the corresponding MxB proteins exhibited >4.5-fold restrictive activity in the context of the WT N-terminus. The deletion of these regions in the context of the Δ1-25 + NLS variant suggested they might still contribute to MxB activity, as these two proteins were largely attenuated for antiviral function (Figure 10C).

## Discussion

MxB was recently discovered to inhibit HIV-1, yet little consensus was found on the stage(s) of infection that was primarily affected. The results reported here shed light on the targeted steps of the viral lifecycle as well as aspects of MxB function that are required for HIV-1 restriction.

### CA is a major determinant of HIV-1 restriction by MxB

Our work confirms that viral CA is a major genetic determinant of MxB restriction. Many individual CA missense mutations, including those of residues on distantly located surfaces of the CA N-terminal domain, rendered the virus resistant to MxB restriction (Figure 2). Similarly, a large set of CA mutations in the N- and C-terminal domains of CA were recently found to confer resistance to MxB [50]. The pattern of CA mutant resistance correlated well with those observed previously for other CA targeting host proteins, such as TRIM5α, CPSF6_358_, and NUP153 (Figure 2E,F and Additional file 1: Figure S1). Although more work would be required to determine why MxB activity did not correlate as strongly with restriction by the artificial Trim-CPSF6_358_ fusion protein, the pattern of Trim-CPSF6_358_ restriction appears generally distinct from those imparted by other factors such as TRIM5α, Trim-NUP153_C_, and the parental non-fused CPSF6_358_ protein [26]. Perhaps the higher-order multimerization that is instilled through the TRIM RING, B-box, and coiled-coil domains [51,52] helps to counteract the brunt of viral changes that otherwise confers resistance to CPSF6_358_.

Our results suggest that a relatively common pleiotropic factor, such as differential CA core uncoating, may simultaneously perturb viral sensitivity to a range of CA-targeting host proteins, including MxB. While this work was in review, Fricke et al. [53] reported that MxB expression increased the level of pelletable CA during acute HIV-1 infection, indicating that the restriction factor alters uncoating through stabilization of incoming viral capsids. The effect caused by CA mutations may be related to MxB-CA binding, as we have observed that MxB-HA in cell extracts can co-sediment *in vitro* with multimerized HIV-1 CA structures [54]. Fricke et al. [53] reported similar co-pelleting between ectopically expressed MxB protein and recombinant CA assemblies *in vitro*.

### MxB determinants required for HIV-1 restriction

Aided by the relatively high sequence conservation between MxA and MxB, we compared genetic determinants of MxB restriction with those previously determined to be required for MxA antiviral activity. Our expanded panel of putative active site mutants supported the dispensability of MxB GTPase activity for HIV-1 inhibition [9,11]. This contrasts with MxA inhibition of Influenza virus, which requires GTP hydrolysis [9]. Concordantly, we found that the various loops extending from the MxB stalk were not critical for antiviral activity. This includes Loop4, which is one of two locations in MxB that exhibits the greatest sequence dissimilarity with MxA. Notably this finding contrasts with the importance of MxA Loop4 for inhibition of Influenza A and Thogotovirus infection [47]. The equilibrium of intracellular MxB multimerization or aggregation likely has an effect on its antiviral activity: the MxB stalk mutants with diffuse localization were less active than the WT protein, with the R455D mutant that exhibited prominent intranuclear staining nearly inactive against HIV-1 (Figure 8). The interpretation of R455D loss-of-function is therefore cautioned by the fact that this mutant may largely be in the wrong place in the cell to exert any potential antiviral activity. Although we have not directly measured MxB protein multimerization, the participation of peptide loops and specific residues (Gly439, Arg455) is inferred from the analogous role of these conserved features in the oligomerization of other dynamin family members [13,48,49].

The other part of MxB with greatest dissimilarity to MxA is the N-terminal region that precedes the first helix of the common BSE. The importance of the first 25 amino acids for antiviral activity was indeed due to its NLS, as the heterologous SV40 NLS conferred full restriction activity to the otherwise dead Δ1-25 construct (Figure 9). Additionally, forced mislocalization of MxB by appending an exogenous NES disrupted antiviral activity, further demonstrating the importance of MxB subcellular localization. In contrast to the first 25 residues, the remaining ~65 residues of the N-terminal stretch contributed to antiviral activity, yet could not be complemented by addition of a heterologous NLS. Thus, particular functions conferred by the peptide sequences in this sub-region appear important. The constructs generally exhibited less antiviral activity when expressed in the context of the Δ1-25 + NLS variant, suggesting a potential interplay between regions within the N-terminal 90 residues of the protein. The sequence present in residues 26–50 were particularly important for MxB activity; this finding is consistent with recent results by Busnadiego et al., who demonstrated individual residues within this region, specially amino acids 37–44, to determine antiviral specificities between MxB proteins isolated from different animal species [50]. Also consistent with our findings is the result that the transfer of MxB residues 1–91 can confer potent anti-HIV-1 activity to human MxA [55]. Although Fricke et al. have highlighted the importance of the N-terminal 25 residues in CA binding [53], additional work is required to more fully characterize the precise role of the unique N-terminal region of MxB for its antiviral mechanism.

### MxB restricts PIC nuclear import and HIV-1 integration

Similar to the results of Kane et al. [11], we found that MxB expression decreased the amount of 2-LTR circles to a level that was intermediate to the decreases observed for HIV-1 integration and virus infection (Figure 4). Concordantly, the extent at which MxB restricted the D64N/D116N IN mutant, a virus whose low level of expression is independent of functional integration, paralleled the 2-LTR circle defect. These observations indicated that a step in the HIV-1 life cycle after nuclear entry was additionally affected by MxB expression. Interestingly, PICs isolated from both the cytoplasm and nucleus of MxB-expressing cells were fully active (Figure 5A), suggesting the defect to integration lies outside the catalytic capacity of the IN enzyme. These results indicate that MxB restriction disrupts multiple nuclear steps to infection, which likely compound to form its potent antiviral activity (Figure 3C,D).

It may at first glance seem counterintuitive that a protein that accumulates in the cytoplasm and at the nuclear rim at steady-state [18] (Figure 1C) can potently inhibit nuclear events. Integration sites from control and MxB-expressing cells were determined to gain insight into the post nuclear entry block to HIV-1 infection. Our data revealed rather dramatic effects of MxB expression on the distribution of integrated proviruses. Significant differences in integration within genes and nearby TSSs and CpG islands were evident (Table 1, Figure 6A, B, and Additional file 3: Figure S3). The ability for HIV-1 to integrate into relatively gene-dense regions of chromatin was additionally affected. Moreover this response seemed similar to those previously noted by depleting cellular import factors such as transportin 3, RANBP2 [45] (Figure 6C,D), or NUP153 [42,56]. These observations seemingly agree with recent research that indicates that HIV-1 PIC nuclear import and integration may be functionally linked [42,45,56,57]. For example, CA point mutations [42,57], in addition to depletion of cellular transport factors [42,45,56], can significantly affect the distribution of HIV-1 integration within chromatin. The tendency for HIV-1 to integrate into host DNA in the vicinity of the nuclear periphery is moreover consistent with these observations [58]. We accordingly speculate that additional investigations into the mechanism(s) of MxB restriction may uncover further novel aspects of HIV-1 PIC nuclear import and integration.

## Conclusions

Our results are consistent with the notion that MxB restricts HIV-1 after DNA synthesis at steps that are coincident with PIC nuclear import and integration. This conclusion was based not only on qPCR analysis of DNA replication intermediates, but also on relative degrees of HIV-1 CA and IN mutant virus sensitivities to MxB antiviral function and results of integration site sequencing. On the host factor side, our results confirm prior reports that GTPase active site residues are largely dispensable for restriction [9,11] and importantly extend these observations to show that various stalk domain loops, which mediate the functional oligomerization of related enzymes, are in large part dispensable for restriction of HIV-1 infection. We additionally confirmed the importance for the N-terminal 25 residues of MxB [11] and extended this finding to show that the heterologous basic-type NLS from SV40 large T antigen fully rescued the restriction activity of the Δ1-25 variant. Approximate quarter-size deletions of the N-terminal 90 residues additionally highlighted an important function within residues 27–50 that is independent of the endogenous NLS within the N-terminal 25 residues of MxB or the added, heterologous SV40 NLS. We therefore conclude that the N-terminal 90 residues of MxB likely contains critical bipartite functional elements for HIV-1 restriction activity.

## Methods

### Cells

HEK293T and HOS cells were cultured in Dulbecco’s modified Eagle’s medium (DMEM) (Invitrogen) supplemented with 10% fetal bovine serum (FBS), 100 U/ml penicillin, and 0.1 mg/ml streptomycin. HOS cells stably transduced by LPCX-based vectors were selected and maintained with 2 μg/ml puromycin.

### Plasmids

Plasmids encoding green fluorescent protein (GFP) reporter HIV-1, SIVmac251, EIAV, FIV, and MLV viral vectors have been described previously [25]. HIV-1 CA, IN, NC, and RT mutations were generated with site-directed mutagenesis of the HIV-1_NL43_-based pHP-dI-N/A packaging plasmid [59] (AIDS Research and Reference Reagent Program [ARRRP]). WT and mutant plasmids were cotransfected with the pHI-Luc transfer vector [25]. Plasmid pLPCX was obtained from Clontech.

DNA sequence encoding human MxB (accession number NM_002463.1) was engineered with a C-terminal HA-tag into the pLPCX-based MLV transduction vector. Mutations were generated using site-directed mutagenesis. The ΔLoop4 mutation was generated by replacing nucleotides corresponding to MxB residues 584 to 615 with those encoding the GAGAG peptide. Stalk Loop1 (residues 440 to 448) and Loop2 (residues 488–497) were replaced with the sequence GSGGSG. The Δ25 mutation was generated by deleting sequences encoding Met1 to Glu25. The NLS Δ25 mutant was created by inserting the SV40 large T antigen NLS sequence (PKKKRKV) subsequent to the initial Met but preceding Asn27. The NES mutant was generated by adding the NES-derived sequence from cAMP-dependent protein kinase inhibitor alpha (LALKLAGLDI) subsequent to the initial Met but preceding Ser2.

### Infection assays and qPCR

MxB-expressing or parental HOS cells (10^4^) seeded onto wells of 48-well plates were infected in duplicate with GFP reporter viruses. Percentages of GFP-positive cells were determined 48 h post infection using a FACSCanto flow cytometer. MxB-expressing or parental HOS cells (2,500) seeded onto wells of 96-well plates were infected in triplicate with luciferase reporter viruses, which were lysed and analyzed 48 h post infection. For qPCR assays, MxB-expressing or parental HOS cells (2 × 10^6^) were infected with 2.5 × 10^7^ RT counts per minute (RTcpm) of HIV-1 luciferase reporter virus in a 10 cm dish in the presence or absence of 20 μM efavirenz (EFV; obtained from ARRRP) to define residual plasmid DNA levels potentially carried over from transfection. After 2 h, cells were washed with phosphate buffered saline (PBS), harvested for the initial time point, and replated into 6 cm plates in the presence or absence of EFV. Cells were collected at additional time points, and DNA was extracted with a QIAamp DNA Mini kit (Qiagen).

QPCR for the accumulation of viral late reverse transcription (LRT) products and 2-LTR-containing circles were performed as previously described [25]. The quantitation of intact 2-LTR circle junctions was performed using primers AE4450 and AE4451 combined with Taqman probe AE2623 (Jxn1; FAM-AAAATCTCTAGCAGTACTGGAAGGGCTAAT-TAMRA), or primers AE4450 and AE5209 (GTGAATTAGCCCTTCCAGTAC) with Taqman probe AE4452 (Jxn2). Values from EFV-treated samples were subtracted from nondrug-treated values. Integration was assessed using nested qPCR as previously described [25].

### Integration site sequencing

Control and MxB-expressing HOS cells (5 × 10^6^) infected with GFP reporter virus for 6 h were washed and then incubated for 5 d to enable dissolution of unintegrated viral DNA prior to DNA isolation using the DNeasy Blood and Tissue Kit (Qiagen). DNA (20 μg) digested overnight with MseI and BglII was purified using the QIAquick PCR Purification Kit (Qiagen). A double-stranded asymmetric linker was made by heating 10 μM oligonucleotide AE5972 (5′-TAGTCCCTTAAGCGGAG/3AmMO/-3′) with 10 μM AE5974 (5′-GTAATACGACTCACTATAGGGCNNNNNCTCCGCTTAAGGGAC-3′) for 2 min at 90°C in 10 mM Tris–HCl, pH 8.0–0.1 mM EDTA, followed by slow cooling to room temperature. The random nucleotides at the center of AE5974 comprise a “serial number”, which was not applicable to this study. Linker DNA (1.5 μM) was ligated with digested cellular DNA (1 μg) overnight at 16°C in four parallel reactions, and the DNAs were pooled and re-purified using the QIAquick PCR Purification Kit. PCRs multiplexed into eight separate samples each contained 1 μg DNA substrate and primers AE5976 and AE5971 in Advantage 2 PCR Buffer. AE5976 (5′-**CAAGCAGAAGACGGCATACGAGAT***CGGTCTCGGCATTCCTGCTGAACCGCTCTTCCGATCT*GTAATACGACTCACTATAGGGC-3′), which was complementary to the linker, additionally contained the Illumina P7 adapter sequence (bold characters) and sequence complementary to the Illumina Paired End read 2 sequencing primer (italics). AE5971 (5′-**AATGATACGGCGACCACCGAGATCTACAC**TCTTTCCC*T*ACACGACGC*T*C*TT*CCGA*T*C*TCTAGT*GAGA*T*CCC*T*CAGACCCTTTTAGTCAG-3′), which contained HIV-1 U5 sequences, also contained the Illumina P5 adapter sequence (bold characters) and a barcode (italics) that was varied among similar primers to track the different samples. The sequence between P5 and the barcode is complementary to the Illumina Read 1 sequencing primer. PCRs were incubated at 94°C for 4 min, followed by six cycles at 94°C for 15 sec, 60°C for 30 sec, and 68°C for 45 sec. Reactions were subsequently cycled 24 times at 94°C for 15 sec, 55°C for 30 sec, and 68°C for 45 sec, which was followed by a final extension for 10 min at 68°C. Pooled PCRs were purified using the QIAquick PCR Purification Kit and sequenced on the Illumina MiSeq platform at the Dana-Farber Cancer Institute Molecular Biology Core Facilities and on the Illumina HiSeq platform at the University of California at Irvine Genomics High Throughput Facility.

### Bioinformatics analysis of integration sites

Representative transcripts from sets of transcripts with identical coordinates yielded 26,251 unique RefSeq human genes from the UCSC Genome Bioinformatics browser [60]; 18,273 genes that yielded overlapping coordinates were omitted from the analyses. The MRC was generated following in silico digestion of the hg19 reference genome with MseI and BglII. CpG island coordinates were downloaded from the UCSC Genome Bioinformatics browser.

LTR sequences were trimmed from each read; duplicates were removed from trimmed reads and the resulting sequences were aligned to hg19 by Blat [61]. Alignments with e values less than 0.05 and that matched starting from the first nucleotide after the LTR were selected. Matches to multiple genomic sequences were removed on the basis of bit scores (differences <0.0001) to identify unique alignments. The start position of the alignment on the positive strand was chosen as the insertion site whereas 4 nucleotides were subtracted from the start position of alignments on the negative strand.

To map insertions relative to TSSs, the distances between insertions and the nearest TSS were calculated and these were summed in 1.25 kb bins. If insertions were upstream of the nearest TSS the distances were plotted with negative numbers. To map insertions relative to CpG islands the inserts were given negative distances if the coordinates of an insertion were less than the coordinates of the nearest CpG. Positive distances were used if the insertions had larger coordinates than that of the nearest CpG. Bedtools was used to calculate the gene densities for each insertion [62].

### Immunofluorescence microscopy

Parental HOS cells or cells stably expressing HA-epitope tagged MxB proteins were cultured on Nunc Lab-Tek II chamber slides (Thermo Scientific). Cells were fixed with 4% paraformaldehyde for 10 min, washed with PBS, and permeabilized with ice-cold MeOH for 10 min. The cells were then blocked with blocking buffer (PBS containing 10% FBS) for 30 min, and stained with 1:300 dilution of anti-HA antibody 16b12 (Covance). After a 30 min wash with blocking buffer, the cells were incubated for 1 h with a 1:1,000 dilution of an Alexa Fluor 555 conjugated goat anti-mouse IgG antibody (Invitrogen) as well as Hoescht 33342 (Invitrogen) at 1 μg/ml. After an additional 30 min wash with PBS, the samples were covered with mounting medium [150 mM NaCl, 25 mM Tris–HCl pH 8.0, 0.5% N-propyl gallate, and 90% glycerol]. The processed samples were analyzed on a Nikon Eclipse spinning disk confocal microscope at the Dana-Farber Cancer Institute Confocal and Light Microscopy core.

### Western blotting

Cells pelleted at 300 × g were resuspended in PBS supplemented with 0.2% NP-40 and 10 U/ml Turbo DNAse (Ambion) and incubated for 30 min on ice. Samples were mixed with protein sample loading buffer to the final concentrations of 62.5 mM Tris–HCl, pH 6.8, 2% sodium dodecyl sulfate, 10% glycerol, 5% 2-mercaptoethanol, and 0.001% bromophenol blue. The samples were heated for 5 min at 100°C, separated on Tris-glycine polyacrylamide gels, and transferred to polyvinylidene fluoride membranes. MxB-HA was detected with a 1:2000 dilution of HRP-conjugated 3F10 antibody (Roche), or a 1:1000 dilution of anti-MxB antibody N-17 sc-47197 (Santa Cruz Biotechnology). Beta-actin was detected with a 1:10,000 dilution of HRP-conjugated antibody clone AC-15 (Sigma). Western blots were developed using ECL prime reagent (GE Healthcare Life Sciences) and imaged with a ChemiDoc MP imager (Bio-Rad) equipped with Image Lab 4.1 software. The amount of MxB-HA or beta-actin signal in each sample was quantitated relative to the level of each signal compared to a matched WT MxB-expressing sample. The MxB expression ratio was calculated by dividing the MxB-HA signal with that of beta-actin, with the level for WT MxB expression set to 1.

### *In vitro* integration assay

The *in vitro* integration activity of PICs isolated from acutely infected cells was determined essentially as previously described [63,64]. In brief, MxB-expressing and control HOS cells (2.1 × 10^7^) plated into three 15 cm dishes were infected the next day with 15 ml of fresh GFP reporter HIV-1 per plate in the presence of 8 μg/ml polybrene for 7 h, at which time cells were harvested for biochemical fractionation. Multiplicity of infection (MOI) was determined two days later by flow cytometry, with an average MOI on MxB-expressing cells of ~0.2 across experiments. PICs isolated from cytoplasmic and nuclear extracts were reacted with plasmid target DNA *in vitro*, and the extent of viral DNA integration was quantified using nested PCR. The first round (27 cycles) amplified covalently linked HIV-plasmid sequences; 1 μl of 100-fold diluted first round PCR product was used in the HIV-1-specific second round qPCR. Values obtained from parallel PCRs that omitted the plasmid-specific primers from the first round of amplification were subtracted from matched experimental samples. Levels of IN-mediated DNA strand transfer activities were normalized to the total level of HIV-1 DNA (qPCR for late reverse transcription products) in each extract.

### Statistical analysis

Correlations between variables were assessed by Spearman rank correlation, and significances of pair-wise differences were calculated by two-tailed Student’s *t*-test, using Prism6 software (Graphpad). Statistical differences between integration site datasets were calculated using R [65].

## Additional files

Additional file 1: Figure S1. Scatterplot comparisons of MxB sensitivity with the indicated CA-mediated host cell determinant. Results of at least 6 independent experiments are expressed as log_10_ averages. Additional file 2: Figure S2. Relative resistance of IN mutant D64N/D116N to MxB restriction over a range of virus inoculum. (A) Levels of WT (two darker shades of grey) and D64N/D116N (NN) IN mutant (lighter shades of grey) infectivity, which were normalized to the WT based on input levels of exogenous RT activity, in control HOS versus MxB-expressing cells. The level of WT infectivity in control cells at the highest multiplicity of infection, which was 25 RT cpm per cell, was set to 100%. (B) Re-plot of panel A to highlight extent of WT (dark grey) versus IN mutant NN (light grey) restriction by MxB at the different multiplicities of infection. The results are the averages of two independent experiments, with error bars denoting standard deviation. Additional file 3: Figure S3. Statistical analysis of HIV-1 integration frequencies surrounding TSSs and CpG islands. (A) *P* value calculations (Fisher’s exact test) for Figure 6A data graphed as three separate curves (control HOS cells versus MRC, blue; MxB-expressing cells versus MRC, green; control versus MxB-expressing cells, red). The purple horizontal line indicates the statistical cutoff value (1.3 = −log_10_(0.05)). The thumbs down sign indicates regions where integration in control HOS cells was significantly disfavored as compared to the corresponding MRC value (relevant bins marked by red cross). The flattening of the WT curve from 2.5 kb to 16.25 kb downstream of TSSs reflects *P* values <2.2 × 10^−308^. (B and C) Expanded views of MxB versus MRC (panel B) and WT versus MxB (panel C) curves from panel A. (D) *P* value analysis of CpG island targeting data from Figure 6B. Other labeling is same as in panel A of this figure. (E and F) Expanded views of panel D green (MxB versus MRC) and red (WT versus MxB) curves. Additional file 4: Figure S4. Scatterplot comparison of WT and mutant MxB-HA expression level with HIV-1 restriction activity. Quantitated levels of mutant MxB-HA expression are expressed relative to WT MxB, which was set to one (vertical dotted line). Blue, single missense mutants; brown, NES and deletion mutant constructs. Expression results are the mean of at least 3 independent experiments, with error bars denoting standard error.
