# Supplementary figures and images for: Host and viral determinants for MxB restriction of HIV-1 infection
Source: Retrovirology. 2014 Oct 25;11:90. doi: 10.1186/s12977-014-0090-z (PMC4213484; doi:10.1186/s12977-014-0090-z)

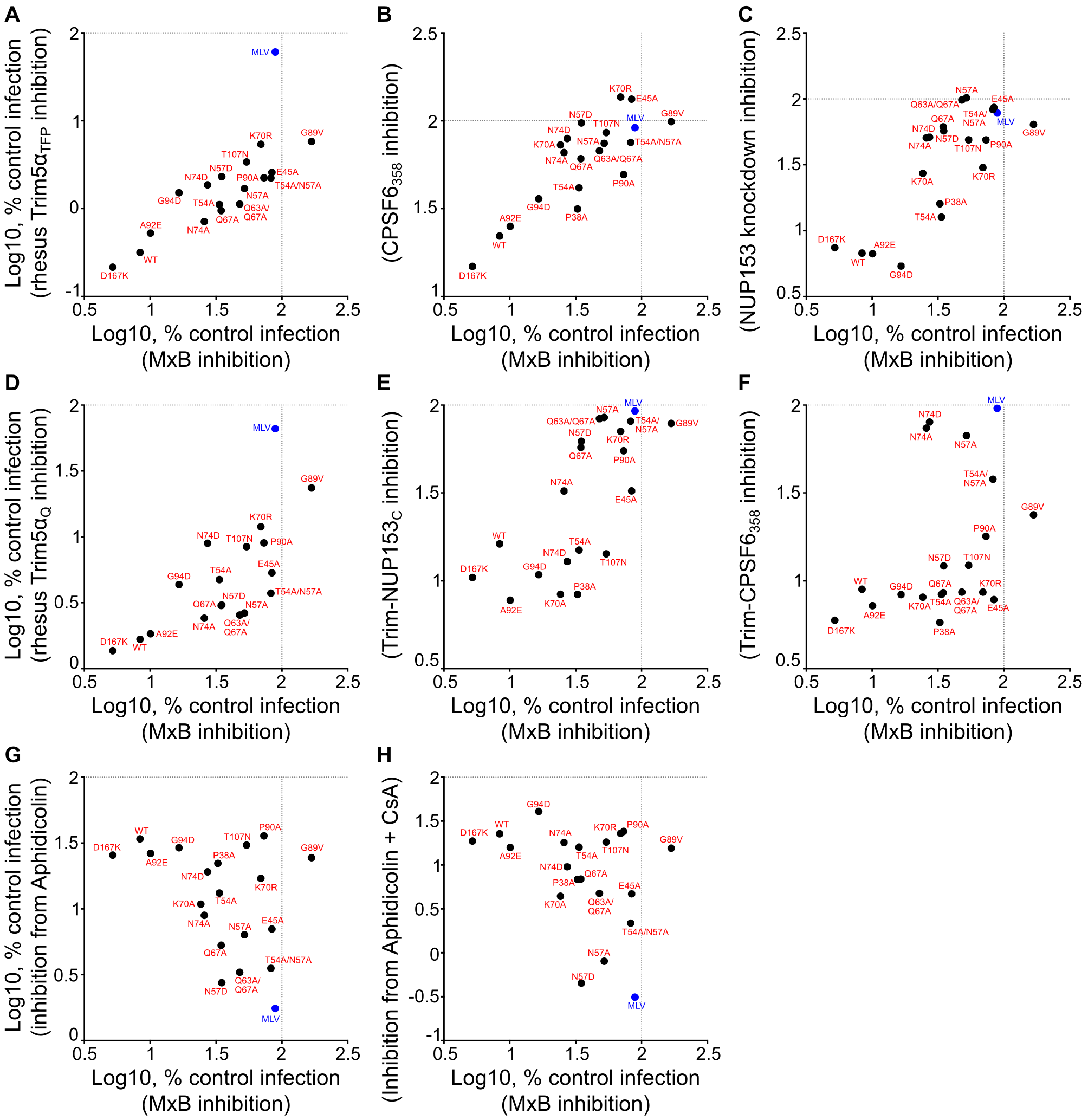

Supplement: Additional file 1: Figure S1. — Scatterplot comparisons of MxB sensitivity with the indicated CA-mediated host cell determinant. Results of at least 6 independent experiments are expressed as log10 averages. [file 12977_2014_90_MOESM1_ESM.png]

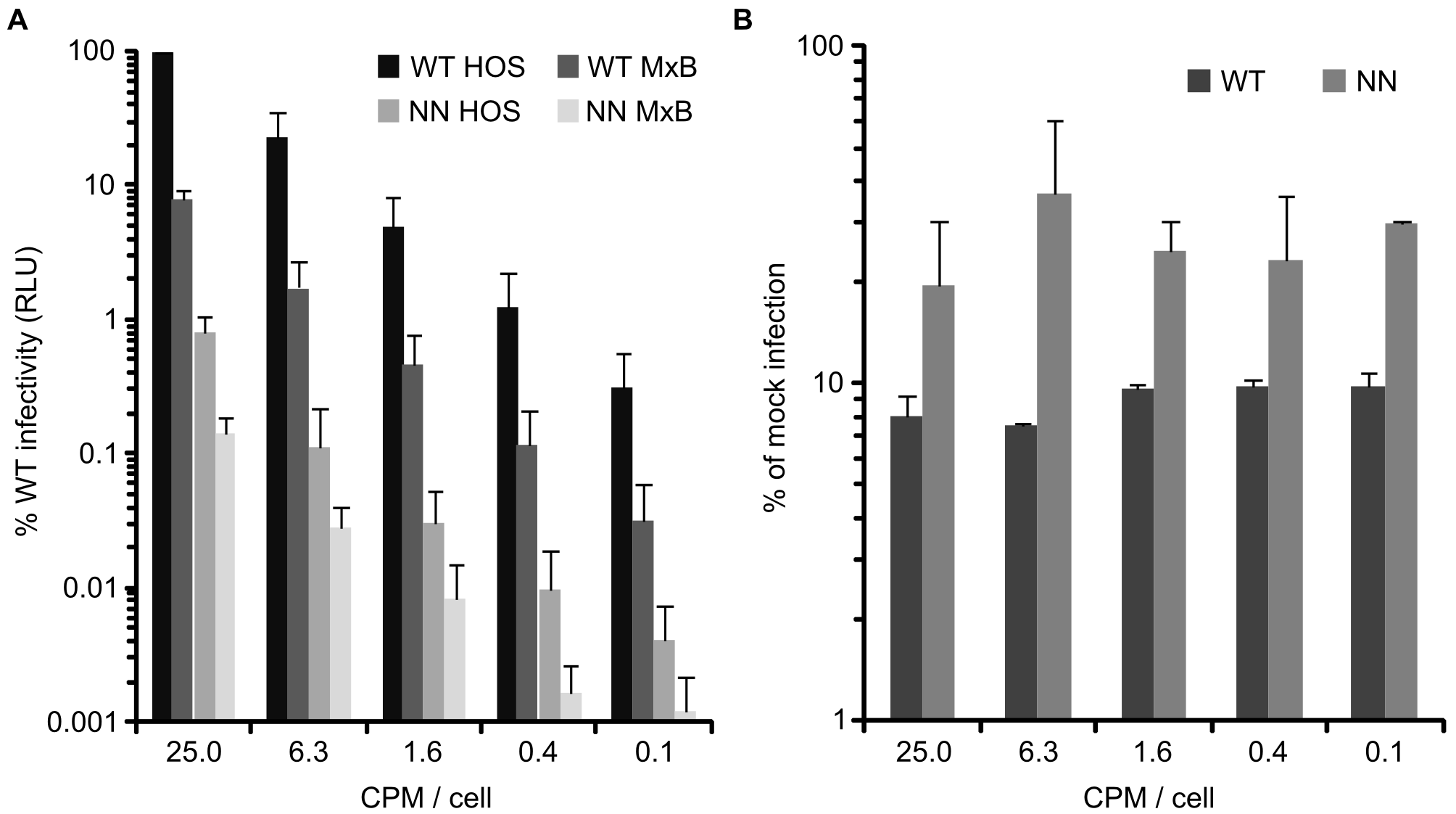

Supplement: Additional file 2: Figure S2. — Relative resistance of IN mutant D64N/D116N to MxB restriction over a range of virus inoculum. (A) Levels of WT (two darker shades of grey) and D64N/D116N (NN) IN mutant (lighter shades of grey) infectivity, which were normalized to the WT based on input levels of exogenous RT activity, in control HOS versus MxB-expressing cells. The level of WT infectivity in control cells at the highest multiplicity of infection, which was 25 RT cpm per cell, was set to 100%. (B) Re-plot of panel A to highlight extent of WT (dark grey) versus IN mutant NN (light grey) restriction by MxB at the different multiplicities of infection. The results are the averages of two independent experiments, with error bars denoting standard deviation. [file 12977_2014_90_MOESM2_ESM.png]

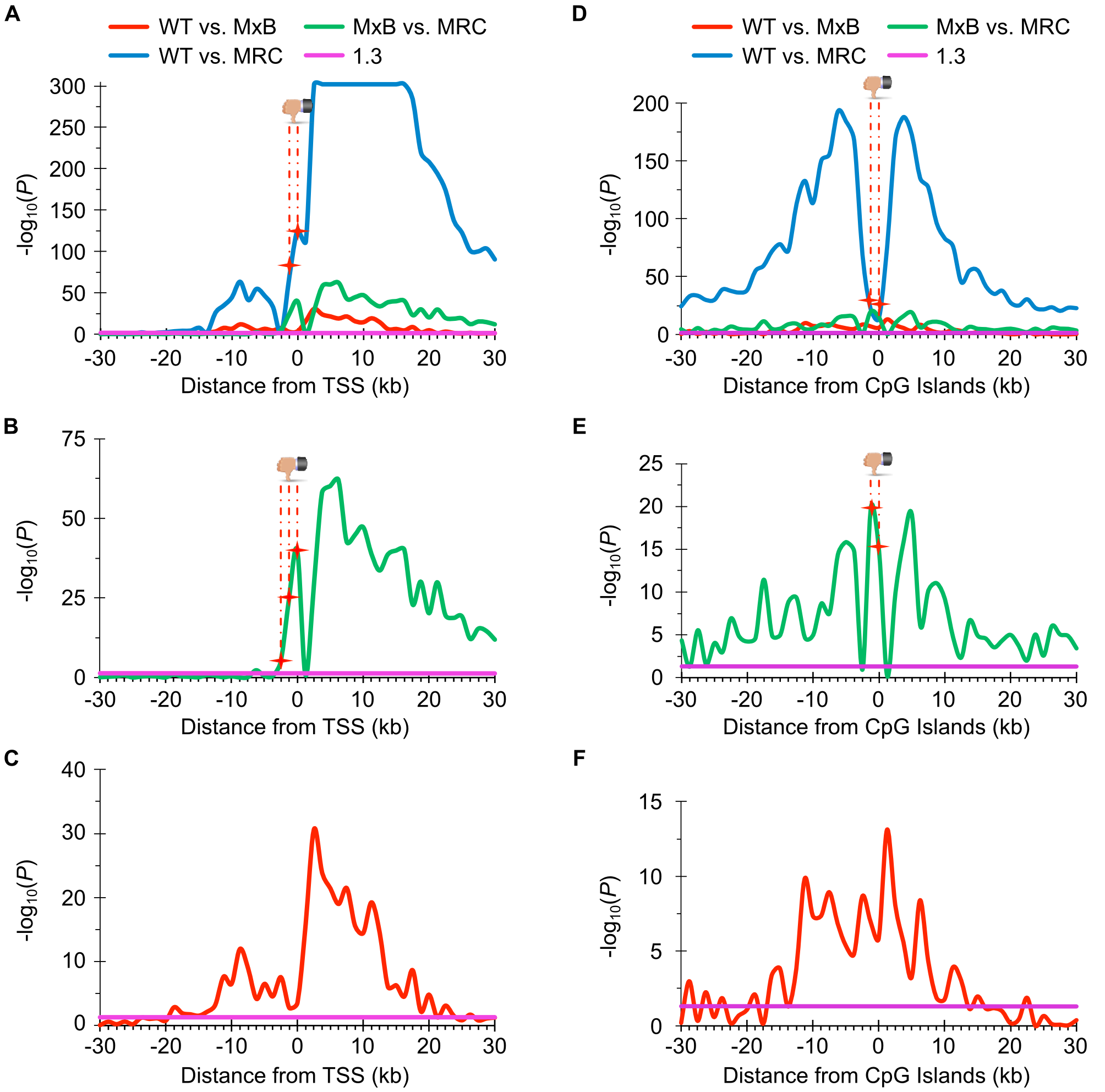

Supplement: Additional file 3: Figure S3. — Statistical analysis of HIV-1 integration frequencies surrounding TSSs and CpG islands. (A) P value calculations (Fisher’s exact test) for Figure 6A data graphed as three separate curves (control HOS cells versus MRC, blue; MxB-expressing cells versus MRC, green; control versus MxB-expressing cells, red). The purple horizontal line indicates the statistical cutoff value (1.3 = −log10(0.05)). The thumbs down sign indicates regions where integration in control HOS cells was significantly disfavored as compared to the corresponding MRC value (relevant bins marked by red cross). The flattening of the WT curve from 2.5 kb to 16.25 kb downstream of TSSs reflects P values <2.2 × 10−308. (B and C) Expanded views of MxB versus MRC (panel B) and WT versus MxB (panel C) curves from panel A. (D) P value analysis of CpG island targeting data from Figure 6B. Other labeling is same as in panel A of this figure. (E and F) Expanded views of panel D green (MxB versus MRC) and red (WT versus MxB) curves. [file 12977_2014_90_MOESM3_ESM.png]

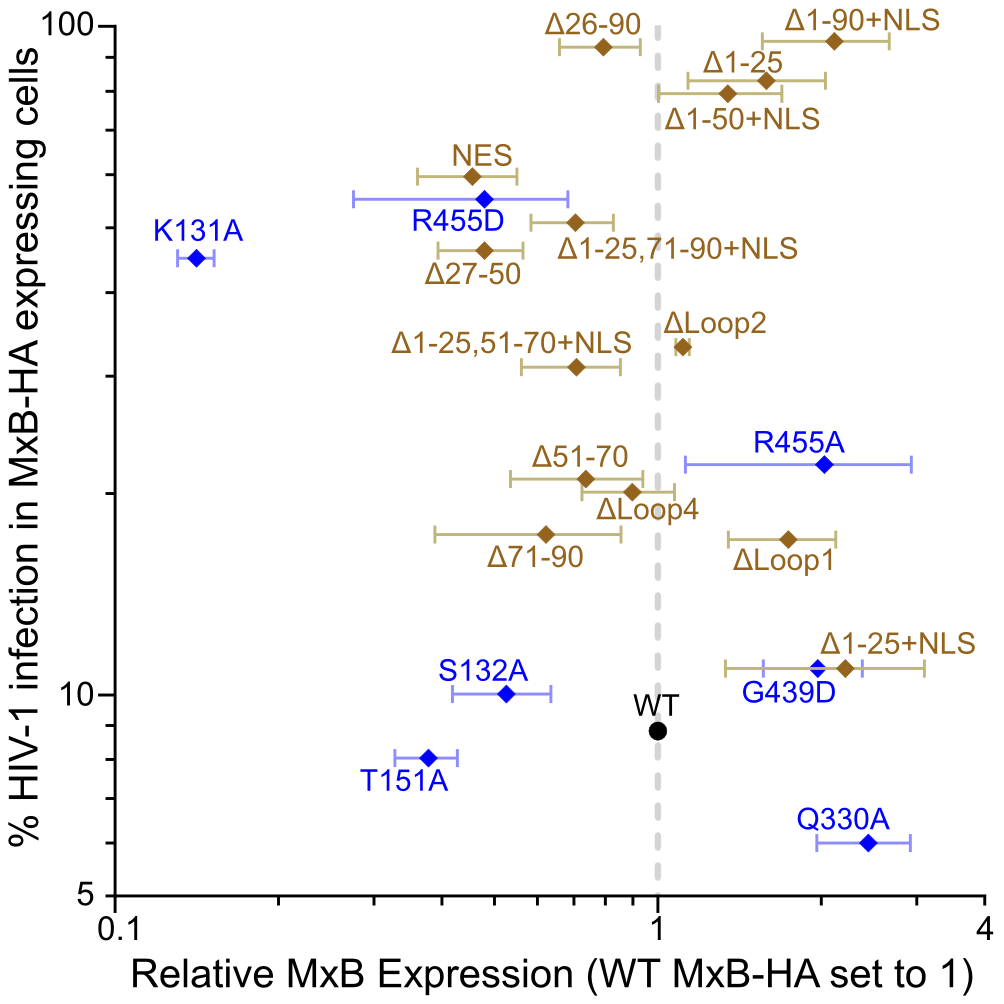

Supplement: Additional file 4: Figure S4. — Scatterplot comparison of WT and mutant MxB-HA expression level with HIV-1 restriction activity. Quantitated levels of mutant MxB-HA expression are expressed relative to WT MxB, which was set to one (vertical dotted line). Blue, single missense mutants; brown, NES and deletion mutant constructs. Expression results are the mean of at least 3 independent experiments, with error bars denoting standard error. [file 12977_2014_90_MOESM4_ESM.png]
